# Temperature-sensitive spinal muscular atrophy-causing point mutations lead to SMN instability, locomotor defects and premature lethality in *Drosophila*

**DOI:** 10.1242/dmm.043307

**Published:** 2020-05-22

**Authors:** Amanda C. Raimer, Suhana S. Singh, Maina R. Edula, Tamara Paris-Davila, Vasudha Vandadi, Ashlyn M. Spring, A. Gregory Matera

**Affiliations:** 1 Curriculum in Genetics and Molecular Biology, University of North Carolina, Chapel Hill, NC 27599, USA; 2 Integrative Program for Biological and Genome Sciences, University of North Carolina, Chapel Hill, NC 27599, USA; 3 Department of Biology, University of North Carolina, Chapel Hill, NC 27599, USA; 4 Gillings School of Global Public Health, University of North Carolina, Chapel Hill, NC 27599, USA; 5 Lineberger Comprehensive Cancer Center, University of North Carolina, Chapel Hill, NC 27599, USA; 6 Department of Genetics, University of North Carolina, Chapel Hill, NC 27599, USA

**Keywords:** *Drosophila* models of human disease, SMN protein, Spinal muscular atrophy, Tudor domain

## Abstract

Spinal muscular atrophy (SMA) is the leading genetic cause of death in young children, arising from homozygous deletion or mutation of the survival motor neuron 1 (*SMN1*) gene. SMN protein expressed from a paralogous gene, *SMN2*, is the primary genetic modifier of SMA; small changes in overall SMN levels cause dramatic changes in disease severity. Thus, deeper insight into mechanisms that regulate SMN protein stability should lead to better therapeutic outcomes. Here, we show that SMA patient-derived missense mutations in the *Drosophila* SMN Tudor domain exhibit a pronounced temperature sensitivity that affects organismal viability, larval locomotor function and adult longevity. These disease-related phenotypes are domain specific and result from decreased SMN stability at elevated temperature. This system was utilized to manipulate SMN levels during various stages of *Drosophila* development. Owing to a large maternal contribution of mRNA and protein, *Smn* is not expressed zygotically during embryogenesis. Interestingly, we find that only baseline levels of SMN are required during larval stages, whereas high levels of the protein are required during pupation. This previously uncharacterized period of elevated SMN expression, during which the majority of adult tissues are formed and differentiated, could be an important and translationally relevant developmental stage in which to study SMN function. Taken together, these findings illustrate a novel *in vivo* role for the SMN Tudor domain in maintaining SMN homeostasis and highlight the necessity for high SMN levels at crucial developmental time points that are conserved from *Drosophila* to humans.

## Abstract (precis)

**Summary:** Using animal models of spinal muscular atrophy, we describe a novel disease mechanism caused by temperature-sensitive protein unfolding/instability of the Tudor domain of SMN.

## INTRODUCTION

Spinal muscular atrophy (SMA) is the leading genetic cause of death in infants and small children, with an incidence of ∼1:7000 live births and a carrier frequency of ∼1:50 (Prior et al., 2010; Sugarman et al., 2012; Vill et al., 2019). This progressive neuromuscular disease is characterized by α-motor neuron degeneration and muscle atrophy, resulting in gradual loss of motor function. SMA symptoms present within a spectrum of disease severity. Left untreated, patients with the most severe form of the disorder are unable to stand or sit upright, and do not survive past 2 years of age (Crawford and Pardo, 1996; Farrar et al., 2017). By contrast, milder forms of SMA are not typically diagnosed until later in life and these patients exhibit mild motor dysfunction, living relatively normal lifespans (Alatorre-Jiménez et al., 2015; Tiziano et al., 2013).

Despite its broad spectrum of severity, SMA is a monogenic disorder that is most commonly caused by homozygous deletion of survival motor neuron 1 (*SMN1*) and a corresponding reduction in the expression of full-length survival motor neuron (SMN) protein. Animal studies have shown that complete loss of SMN protein results in death *in utero* (Schrank et al., 1997); however, the presence of a paralogous gene in humans, *SMN2*, allows for the survival of affected individuals past birth (Coovert et al., 1997). The coding region of *SMN2* is identical to that of *SMN1*, except for five non-polymorphic nucleotide differences, one of which causes skipping of exon 7 during splicing in approximately 90% of *SMN2* pre-mRNAs (Lorson et al., 1999). Transcripts produced by this alternative splicing event are translated into a truncated version of SMN protein (SMNΔ7) and are quickly degraded by the proteasome (Gray et al., 2018; Lorson et al., 1998). The remaining fraction of full-length transcripts (∼10%) encodes full-length SMN that is identical to protein produced by *SMN1*. In humans, *SMN2* is located on chromosome 5q within a highly dynamic genomic region that is prone to both duplications and deletions (Lefebvre et al., 1995). This has led to significant *SMN2* copy number variation in the population (Butchbach, 2016; Carpten et al., 1994; Courseaux et al., 2003). Complete loss of *SMN2* has no phenotypic effect in healthy individuals; however, in SMA patients, *SMN2* is the primary genetic modifier of disease severity (Feldkötter et al., 2002; Lefebvre et al., 1997; Velasco et al., 1996). Higher *SMN2* copy number produces increased levels of full-length SMN protein, which corresponds to later disease onset and milder symptoms. Although the precise molecular etiology of SMA remains unclear, overwhelming evidence shows that reduced SMN protein levels cause the disease (Ahmad et al., 2016; Briese et al., 2005; Chaytow et al., 2018; Deguise and Kothary, 2017; Li et al., 2014).

The importance of SMN protein levels is further evidenced by the fact that the mechanism of action for both US Food and Drug Administration (FDA)-approved treatments currently available for SMA, Spinraza (nusinersen) and Zolgensma (onasemnogene abeparvovec), aim to increase SMN protein levels (Sumner and Crawford, 2018). Although these treatments have dramatically improved the prognosis of SMA patients, there are limitations to the therapies that could be addressed using combinatorial therapies (Gidaro and Servais, 2019; Ramos et al., 2019; Sumner and Crawford, 2018). For example, it remains to be seen whether these treatments will remain effective over time and into adulthood, or if the patients might develop symptoms later in life. Additionally, given the general housekeeping function of SMN in the biogenesis of spliceosomal small nuclear ribonucleoproteins (snRNPs) (Matera and Wang, 2014), long-term treatment of the central nervous system might reveal deficits in peripheral tissues over time. Thus, a multi-pronged approach to precisely control SMN levels and function across tissues is more likely to prevent SMA disease progression throughout a patient's lifetime.

**Fig. 1.**
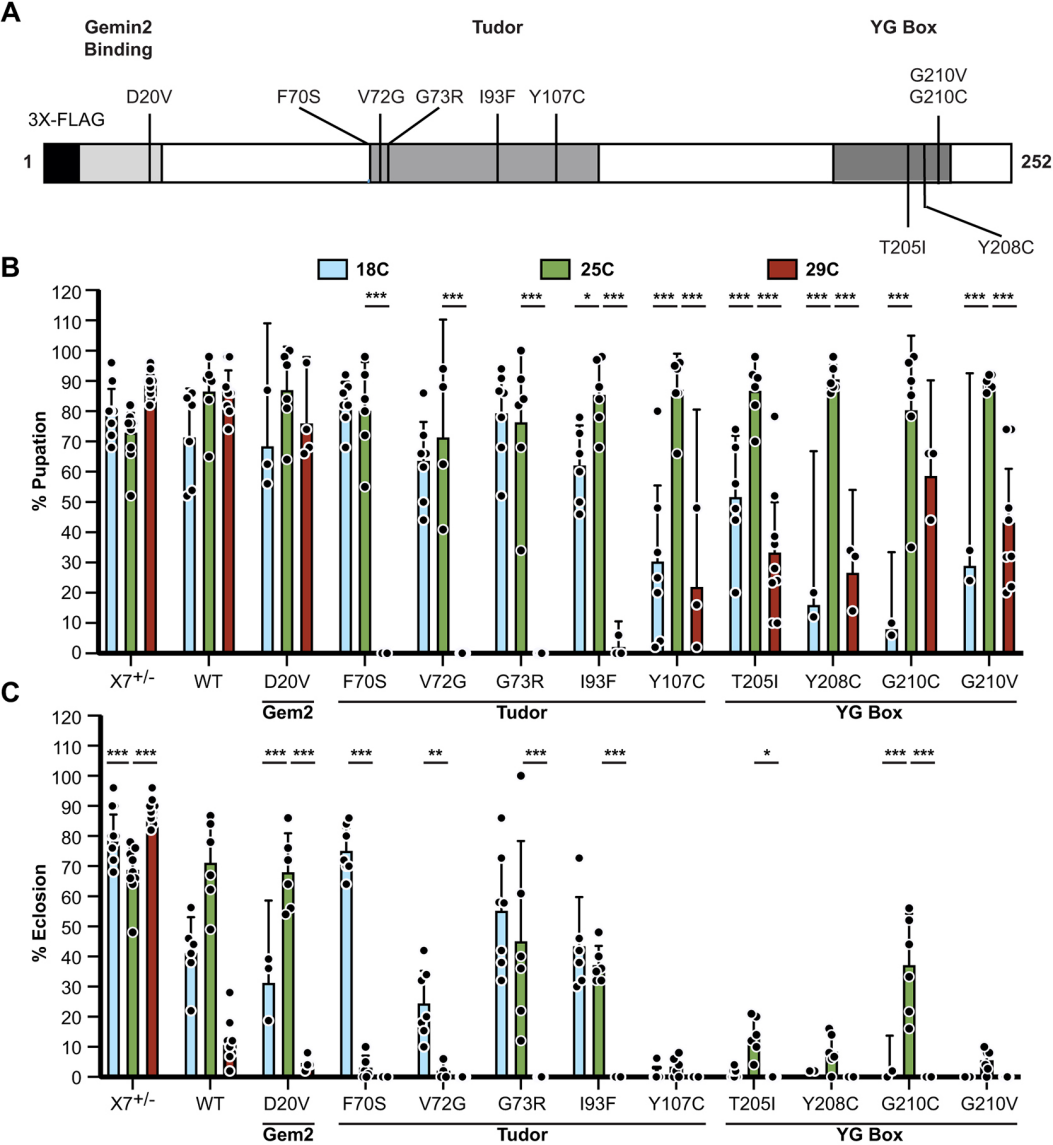
**Effects of temperature limits on viability of SMN patient-derived missense mutant lines.** (A) Diagram of the SMN protein showing orthologous SMA patient-derived missense mutations in *Drosophila* SMN. Schematic of *Drosophila* SMN transgene construct, which includes an N-terminal 3X-FLAG tag. Note that the construct also contains a 1.7 kb upstream sequence that includes the native *Smn* promoter and a 0.4 kb downstream flanking region (not shown). (B,C) Viability assays for the wild-type and mutant *Smn* transgenic lines, measured as the fraction of larvae reaching the pupal (B) and adult (C) stages when raised at either the standard culturing temperature of 25°C (green), at 18°C (light blue) or at 29°C (red). X7^+/–^ animals are heterozygotes that contain one endogenous copy of *Smn*, whereas WT are animals that contain one wild-type copy of the *Flag-Smn* transgene in an otherwise null background. Mutants are organized by their location within the three functional domains of SMN: Gemin2, Tudor or YG box. The number of larvae for each genotype-temperature combination ranged from 100 to 420 animals. Larvae were split into vials of ∼50 animals and viability of each vial was measured as a separate replicate (displayed as data points). Error bars represent mean ±95% c.i. Adjusted *P*-value was calculated using two-way ANOVA and Tukey's multiple comparisons test. *P<0.05, **P<0.01, ***P<0.001. Sample size (number of larvae): X7^+/−^ 18°C (500), 25°C (500), 29°C (500); WT 18°C (139), 25°C (400), 29°C (394); D20V 18°C (128), 25°C (400), 29°C (200); F70S 18°C (150), 25°C (400), 29°C (130); V72G 18°C (113), 25°C (400), 29°C (300); G73R 18°C (122), 25°C (400), 29°C (400); I93F 18°C (144), 25°C (400), 29°C (150); Y107C 18°C (131), 25°C (400), 29°C (150); T205I 18°C (123), 25°C (400), 29°C (420); Y208C 18°C (100), 25°C (400), 29°C (150); G210C 18°C (100), 25°C (400), 29°C (150); G210V 18°C (121), 25°C (400), 29°C (394).

Although most SMA patients carry a homozygous deletion of *SMN1*, 5% of those affected are heterozygous, harboring a deletion of *SMN1* over a small indel or missense mutation (Lefebvre et al., 1995; Wirth, 2000). To better understand how *SMN1* missense mutations contribute to disease, our laboratory has developed *Drosophila* as an SMA model system. Previously, we generated an allelic series of transgenic fly lines that express SMA-causing point mutations in an otherwise *Smn* null mutant background (Praveen et al., 2012, 2014). These animals express FLAG-tagged wild-type or mutant SMN from the native *Smn* promoter (Fig. 1A) and have been used to study SMA phenotypes at behavioral, physiological and molecular levels (Garcia et al., 2013, 2016; Gray et al., 2018; Praveen et al., 2014; Spring et al., 2019).

SMN contains three conserved regions, including the N-terminal Gemin2-binding motif, the C-terminal YG box self-oligomerization module and the centrally located Tudor domain. The presence of disease-causing mutations within each of these three regions demonstrates the importance of each domain to SMN function. Previous work in *Drosophila* and other models has demonstrated a functional role for the YG box in targeting SMNΔ7 for degradation by the proteasome (Cho and Dreyfuss, 2010; Gray et al., 2018). By contrast, very little is known about the effect of the SMN Tudor domain on SMN protein levels. The canonical function of a Tudor domain is to bind to methylated arginine or lysine residues, thereby modifying the activity or function of the target protein (Pek et al., 2012). In the context of SMN, the Tudor domain binds dimethylated arginine residues on Sm proteins (Brahms et al., 2001; Bühler et al., 1999). This interaction assists in the assembly and formation of Sm-class snRNPs (Gonsalvez et al., 2007, 2008; Meister et al., 2001; Pellizzoni et al., 2002). Patient data, together with *in vitro* and *in silico* studies, indicate that certain Tudor domain mutations affect SMN protein levels, but the mechanism underlying this phenomenon remains unclear, especially *in vivo* (Hossain and Hosen, 2019; Li, 2017; Sneha et al., 2018; Takarada et al., 2017; Tripsianes et al., 2011).

Here, we present evidence that point mutations in the SMN Tudor domain are temperature sensitive relative to the wild-type protein, destabilizing SMN at high temperatures. Additionally, we demonstrate that this added degree of instability reduces SMN protein levels sufficiently to affect SMA-related phenotypes such as organismal viability, larval locomotion and adult longevity. The temperature-sensitive nature of these mutations also provides a useful experimental system in which to study how changes in SMN protein levels affect molecular and physiological processes across animal development. Collectively, the results expand our understanding of the mechanisms that govern not only SMN protein stability but also SMA etiology.

## RESULTS

### SMN Tudor domain mutants are temperature sensitive

Previous work using SMA patient-derived *Smn* missense mutations, modeled in the fly, has produced robust and reproducible findings. In one or two instances, however, we noticed inconsistencies in the overall viability of a given fly line that could not be attributed to normal biological noise. For example, in Praveen et al. (2014), the *Smn^F70S^* mutation line (hereafter F70S) displayed a relatively mild phenotype, with an eclosion frequency similar to that of the *Smn^WT^* transgene (hereafter WT). By contrast, Spring et al. (2019) reported a rather severe viability defect for this same F70S line. The husbandry conditions used in each study were slightly different: the experiments in the earlier work were performed at room temperature (∼22°C), whereas in subsequent experiments animals were kept at a constant 25°C. In addition, we qualitatively observed that certain *Smn* mutant lines displayed a dramatic decrease in viability at 29°C compared with those cultured at 25°C. These two observations led us to characterize the mechanism underlying this sensitivity, as determinants of SMN function could be of translational value to SMA patients.

We examined the effects of temperature on the viability of eleven different SMA patient-derived mutant lines (Fig. 1A) at the two temperature extremes of *Drosophila* husbandry, 18°C and 29°C, as well as at the standard condition of 25°C. Viability of each transgenic line was calculated as the fraction of animals that either pupated (percentage pupation, larval-to-pupal transition) or eclosed (percentage eclosion, pupal-to-adult transition) (Fig. 1B,C). A majority of our SMA models displayed the expected trend, with the highest viability observed at 25°C and reduced viability at the extremes. By contrast, all but one of the SMN Tudor domain mutant (TDM) lines displayed an inverse correlation between viability and temperature. That is, lower temperatures increased viability in TDMs relative to 25°C, whereas higher temperatures decreased viability (Fig. 1B,C). The decrease in pupation frequency for the TDMs was dramatic and statistically different from the moderate decrease observed for mutations in other regions of SMN (Fig. 1B). Similarly, only the TDM lines displayed increased eclosion frequencies at 18°C compared with 25°C (Fig. 1C). Collectively, these data indicate that the F70S, V72G, G73R and I93F mutations in the SMN Tudor domain are temperature-sensitive alleles.

### SMN TDMs display SMA-related phenotypes in response to small changes in temperature

**Fig. 2.**
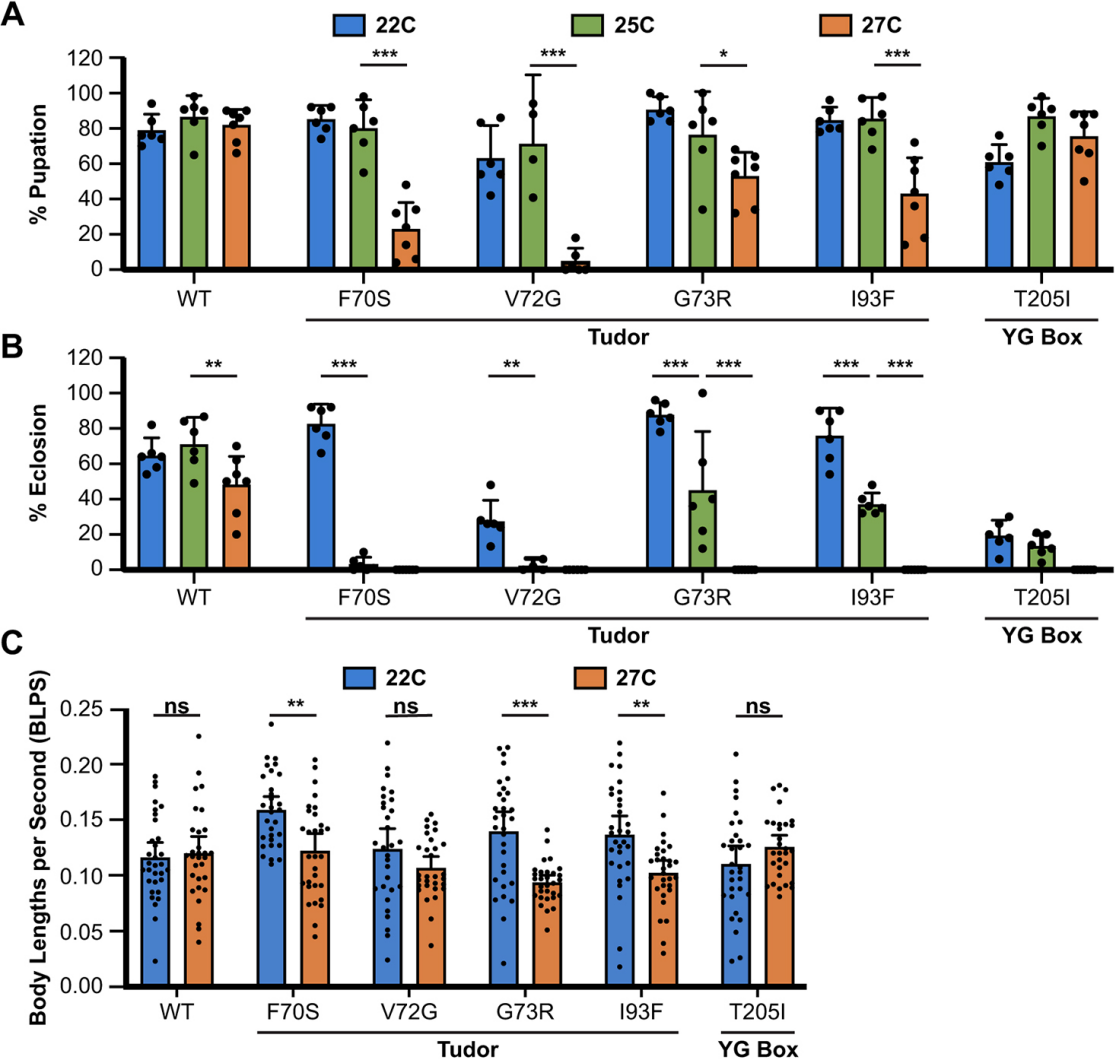
**Effects of small temperature changes on SMN Tudor mutant viability and locomotor function.** (A,B) Viability assay of flies expressing a wild-type *Smn* transgene, one of four Tudor domain mutations (F70S, V72G, G73R or I93F) or a YG box domain mutation (T205I). Viability is measured as the fraction of larvae that reach the pupal (A) and adult (B) stages, while being raised at either 25°C (green), a slightly cooler temperature (22°C, blue) or a slightly warmer temperature (27°C, orange). The number of larvae for each experimental group ranged from 150 to 400 animals. Larvae were split into vials of ∼50 and the viability of each vial was treated as a separate replicate. The 25°C viability data are the same data from Fig. 1C,D and are included for ease of comparison. Error bars represent mean±95% c.i. Adjusted *P*-values calculated using Tukey's multiple comparisons test: *P<0.05, **P<0.01, ***P<0.001. (C) Locomotion assays of wandering 3rd instar larvae of the same genotypes described in A and B raised at 22°C (blue) or 27°C (orange). A total of 30-35 larvae were assayed per condition. Locomotion was measured in body lengths per second (BLPS), which measures the larva's speed in relationship to its body size. Error bars represent mean±95% c.i. *P*-values were calculated using Student's *t*-test: ns, not significant (*P* >0.05); ** *P*<0.01, *** *P*<0.001. For A and B, sample size (number of larvae): WT 22°C (150), 25°C (400), 27°C (350); F70S 22°C (150), 25°C (400), 27°C (350); V72G 22°C (150), 25°C (400), 27°C (300); G73R 22°C (150), 25°C (400), 27°C (350); I93F 22°C (150), 25°C (400), 27°C (350); T205I 22°C (150), 25°C (400), 27°C (350). (C) Sample size (number of larvae): WT 22°C (32), 27°C (30); F70S 22°C (30), 27°C (30); V72G 22°C (30), 27°C (30); G73R 22°C (31), 27°C (30); I93F 22°C (31), 27°C (30); T205I 22°C (31), 27°C (30).

We next examined the effect of minor changes in temperature by raising animals at 22°C and 27°C. The WT and T205I (YG box) mutant lines were used as controls. Although the changes in temperature were relatively small (±2-3°C), the effects on TDM viability were substantial (Fig. 2A,B). The WT and T205I control lines display some variability in their pupation and eclosion frequencies. By contrast, although a fraction of all the TDMs do manage to pupate at all three temperatures, they again displayed a large decrease in viability that inversely correlates with temperature.

Having established the temperature-sensitive nature of the TDMs, we next sought to examine more SMA-related phenotypes, such as locomotor function. Although the temperature extremes, 18°C and 29°C, showed the most drastic changes in viability, certain limitations make these temperatures suboptimal for analyzing *Drosophila* larval locomotion. Proper developmental staging of larvae in many assays is crucial so that changes observed between wild type and TDMs are solely attributed to the mutation and not different developmental stages (Garcia et al., 2013). Because TDMs raised at 25°C die during pupation, the wandering 3rd instar larval stage is one of the most developmentally significant and technically tractable stages to use when performing larval assays. However, at 29°C, most TDMs do not reach the wandering 3rd instar, and viability of the WT transgenic line is markedly affected at both 18°C and 29°C. By contrast, culturing at 22°C and 27°C ameliorates these problems.

Larval locomotion assays were carried out on animals raised at 22°C and 27°C, as described previously (Spring et al., 2019). Crawling speed was expressed in terms of body lengths per second (BLPS), which provides a comparable measure of larval speed regardless of body size (Fig. 2C). Larvae expressing either WT or T205I SMN displayed no significant changes in locomotion when raised at 22°C versus 27°C. By contrast, TDM animals raised at 22°C showed significantly improved locomotor function compared with their counterparts raised at 27°C (Fig. 2C). These results highlight the fact that locomotor phenotypes are exacerbated specifically in the TDM lines raised at elevated temperatures.

### Reduced SMN levels caused by instability underlie TDM sensitivity to temperature

**Fig. 3.**
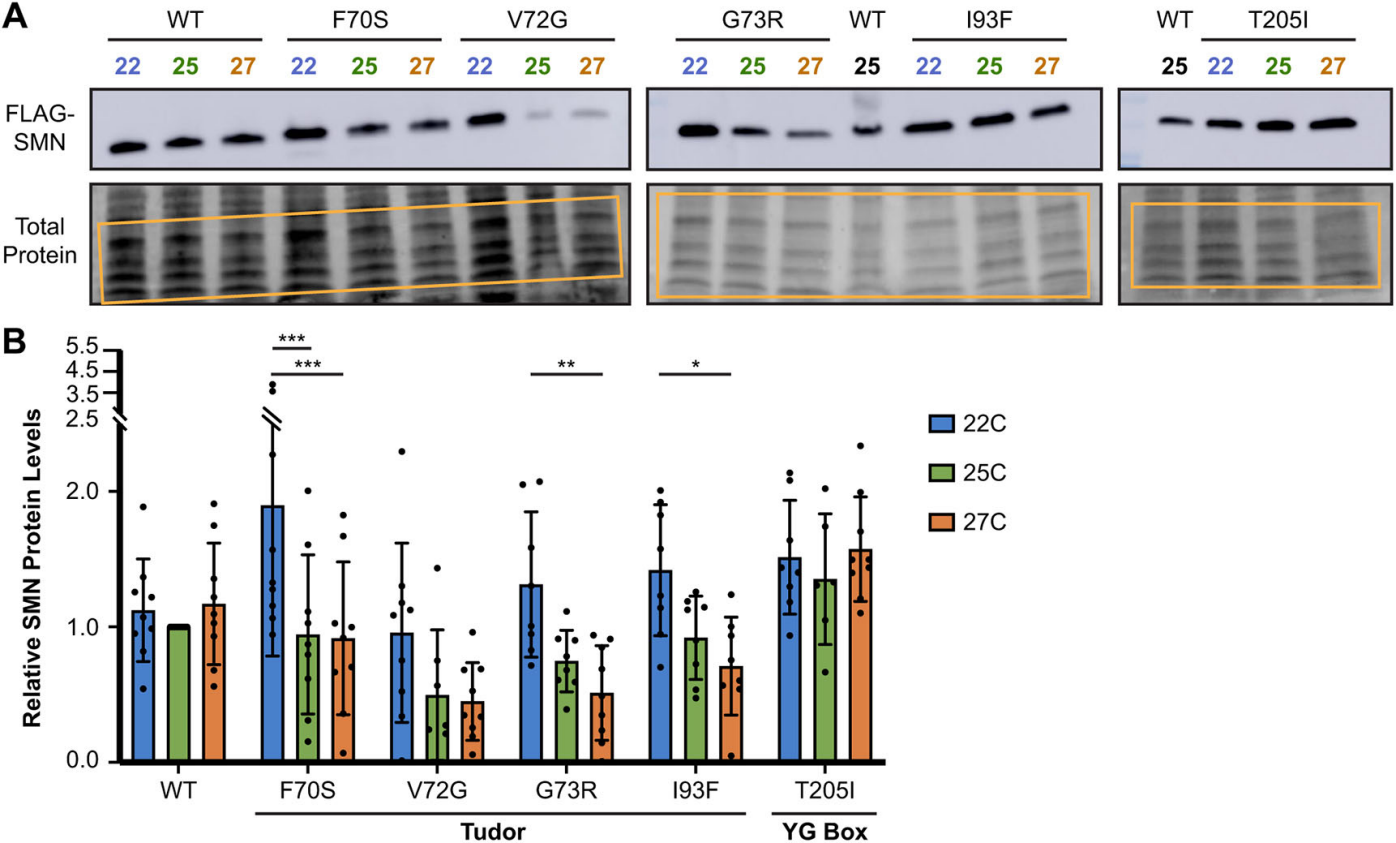
**Effects of small temperature changes on SMN protein levels in Tudor domain mutants.** (A) Representative western blots of wandering 3rd instar larvae from wild type, Tudor domain mutants and YG box domain mutants (T205I) raised at 22°C (blue), 25°C (green) or 27°C (orange) (eight to 12 larvae per sample). Protein was visualized using an HRP-conjugated primary antibody that recognizes the 3X-FLAG tag on the N terminus of the SMN transgenic construct. (B) Quantification of western blot biological replicates represented in A. Each sample contained ten wandering 3rd instar larvae, each genotype-temperature combination contains seven to nine samples. Total protein was used as a loading control to standardize SMN levels (see Fig. S1). SMN levels for each sample were normalized to the ‘WT 25’ SMN protein levels for their respective replicate. Error bars represent mean±95% c.i. The adjusted *P*-value was calculated using two-way ANOVA and Tukey's multiple comparisons test: *P<0.05, **P<0.01, ***P<0.001. Sample size (replicates): WT 22°C (9), 25°C (9), 27°C (9); F70S 22°C (9), 25°C (9), 27°C (9); V72G 22°C (9), 25°C (7), 27°C (9); G73R 22°C (8), 25°C (8), 27°C (9); I93F 22°C (8), 25°C (8), 27°C (8); T205I 22°C (9), 25°C (7), 27°C (9).

Small perturbations in SMN protein levels are known to cause stark changes in patient disease severity (Lefebvre et al., 1997). We therefore evaluated SMN protein levels as a potential cause of the temperature sensitivity observed in the TDMs. SMN point mutation lines bearing a single copy of the *Smn* transgene were raised at 22°C, 25°C or 27°C, and the SMN protein levels of wandering 3rd instar larvae were measured by western blotting. Band intensities were then normalized against the total amount of protein in each sample. Representative blots showing SMN protein levels in the WT and mutant larvae raised at the three different temperatures are shown in Fig. 3A. SMN levels in WT and T205I mutant animals remain relatively constant, regardless of temperature. By contrast, SMN levels are significantly decreased in TDM lines raised at higher temperatures. Quantification of multiple biological replicates (Fig. 3B) confirms that the trend of decreasing SMN protein levels holds for nearly all of the TDMs. Note that the very low levels of SMN in the V72G mutants raised at 25°C and 27°C are close to the detection limit, so the trend is less visible in that line. Collectively, these data indicate that temperature sensitivity of the TDMs is driven by a reduction in SMN protein levels.

**Fig. 4.**
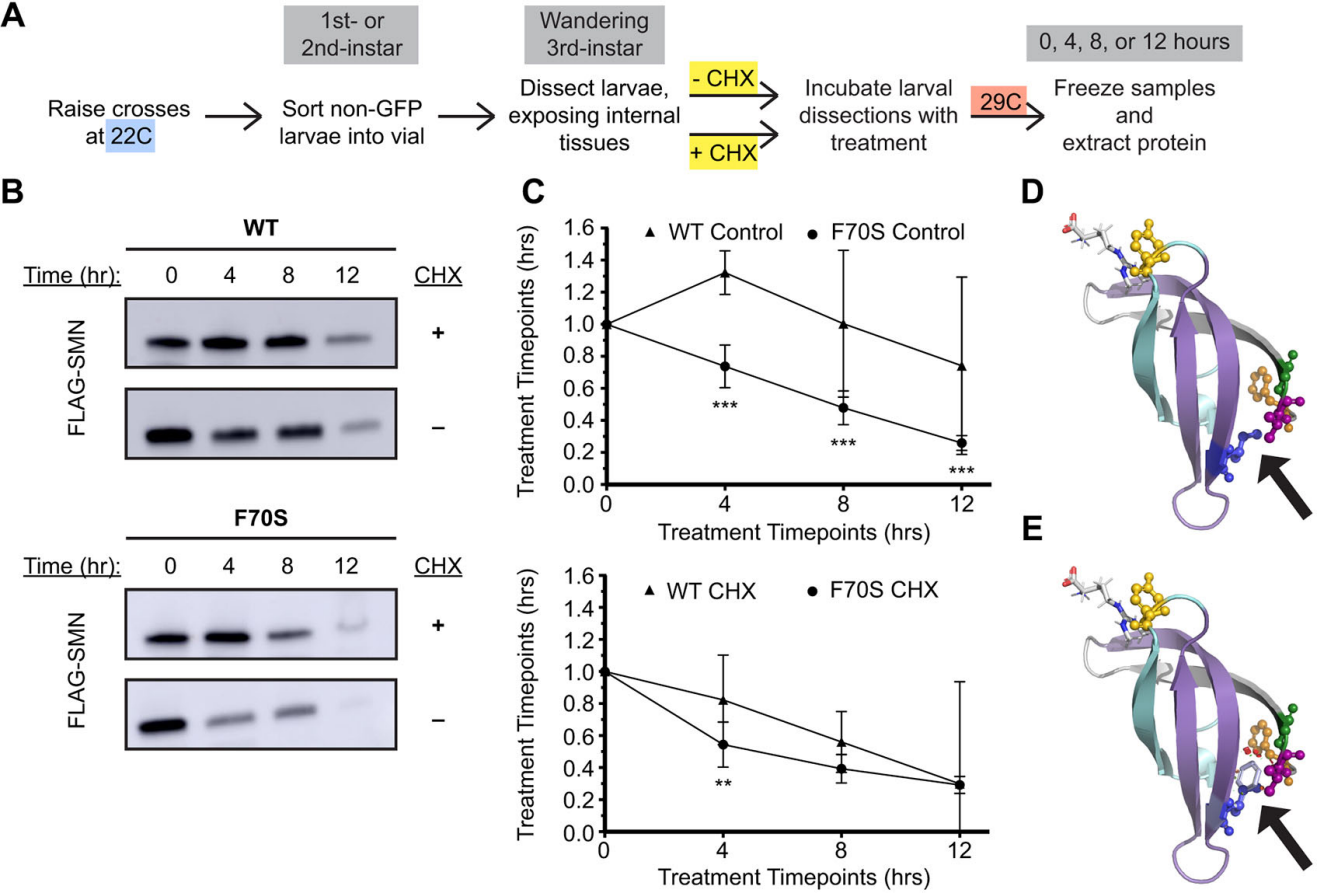
**Cycloheximide experiments measure SMN protein stability in F70S Tudor domain mutants.** (A) Schematic of CHX experiment, showing treatment types, time points, and treatment temperature. (B) Representative western blots of WT and F70S mutant wandering 3rd instar larvae in the presence of CHX over 12 h, ten dissected larvae per sample. The control treatment contained Schneider's medium, while the CHX treatment contained Schneider's medium and CHX. All blots show FLAG-SMN levels, total protein used for standardization not shown. (C) Quantification of western blots represented in B; three to nine samples were analyzed per condition. ‘Control’ represents the medium-only treatment; ‘CHX’ represents the medium and CHX treatment. All SMN protein levels were standardized using total protein and relative to ‘0 h’ SMN levels of each genotype. (D,E) Protein structure model of *Drosophila* SMN Tudor domain with bound dimethylated arginine (red-white-blue stick model, upper left) (generation of the model is described in the Materials and Methods section). (D) Wild-type SMN Tudor domain with residues of interest highlighted by ball-and-stick structures (F70 orange, V72 magenta, G73 green, I93 blue). (E) Same structure as D, but with I93F overlay (silver, black arrow). Steric clash is shown by red circles. This is the most common of four conformations for I93F, but all four conformations experience steric clash. Error bars represent mean±95% c.i. Adjusted *P*-values calculated using Tukey's multiple comparisons test: **P<0.01, ***P<0.001. Sample size (replicates): WT control 0 h (9), 4 h (4), 8 h (4), 12 h (4); F70S control 0 h (9), 4 h (4), 8 h (4), 12 h (4); WT CHX 0 h (9), 4 h (8), 8 h (8), 12 h (3); F70S CHX 0 h (9), 4 h (9), 8 h (8), 12 h (7).

To directly measure the relative stabilities of WT and TDM SMN proteins in the absence of new protein synthesis, we carried out an *ex vivo* protein stability assay similar to the one described by Deliu et al. (2017). Wandering 3rd instar WT and F70S animals (raised at 22°C) were dissected and then the larval filets were incubated in cell culture medium at 29°C in the presence or absence of cycloheximide (CHX) over a time course of 12 h (Fig. 4A). The F70S mutant was chosen for this assay because it displayed a pronounced temperature-sensitive phenotype. Samples were then analyzed for SMN protein levels by western blotting, as shown in Fig. 4B. Puromycin was used as a secondary control to confirm that protein synthesis was effectively stalled in the presence of CHX (Deliu et al., 2017; Fig. S1). In untreated WT samples, SMN levels show a mild reduction from 0 to 12 h post-exposure; however, in the presence of CHX, WT protein levels decrease consistently over the time course, illustrating the natural levels of SMN degradation during this time frame. By contrast, the F70S mutation causes SMN levels to decrease more rapidly (Fig. 4B). Multiple replicates of each genotype at each time point verify these trends (Fig. 4C), confirming that the F70S TDM is significantly less stable than its WT counterpart. These data provide the first evidence to show that SMN Tudor domain mutations trigger protein instability *in vivo*, providing molecular insight into mechanisms by which these point mutations can cause SMA. When modeling the *Drosophila melanogaster* Tudor domain sequence onto the *Homo sapiens* structure, most of the SMA-causing mutations occur in the same spatial region (Fig. 4D) and those mutations appear to cause steric clashes (Fig. 4E). Additionally, the temperature-sensitive nature of these alleles provides a powerful tool for temporally regulating SMN protein levels throughout development and lifespan.

### High levels of SMN are not required during normal larval development but are essential for metamorphosis into adults

Numerous studies in SMA patients and animal models have shown that high SMN levels are vital during the early stages of development (Govoni et al., 2018; Jablonka and Sendtner, 2017; Ramos et al., 2019). Consistent with this idea, we previously showed that null mutations in *Drosophila Smn* result in developmental arrest and early larval lethality (Rajendra et al., 2007; Shpargel et al., 2009; Praveen et al., 2012; Garcia et al., 2013). Maternally deposited SMN is exhausted shortly after the 1st larval instar (Praveen et al., 2012), but a detailed analysis of SMN levels during later stages of development has not been performed. We therefore mined transcriptomic, proteomic and chromatin packaging databases to analyze expression from the *Smn* locus over developmental time. Furthermore, we exploited the temperature sensitivity of TDMs to help determine the requirements for high levels of SMN during larval, pupal and adult stages of *Drosophila* development.

**Fig. 5.**
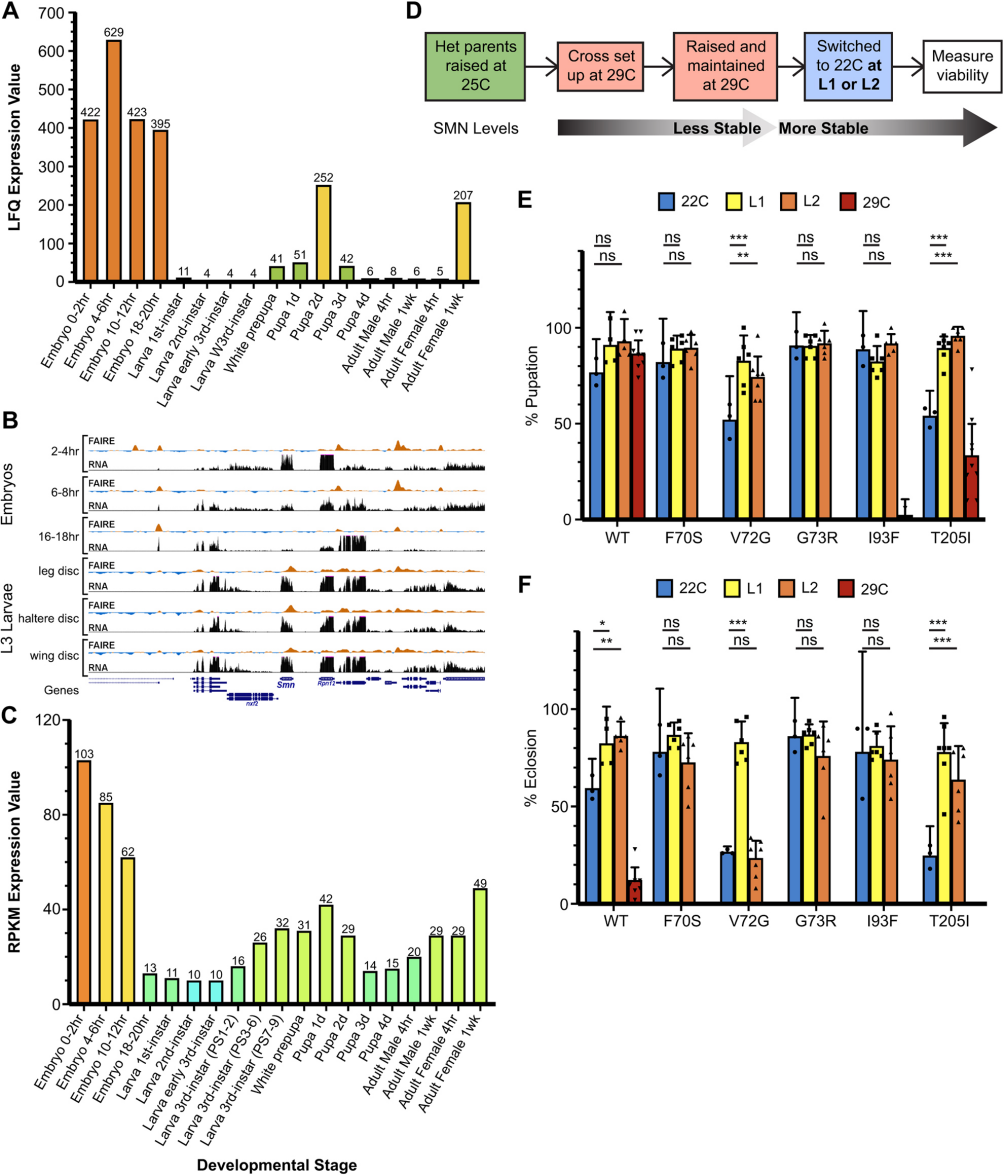
**Non-permissive-to-permissive temperature-shift viability assays during early larval development.** (A) Proteomic analysis of SMN levels throughout *Drosophila* developmental stages, data mined from Casas-Vila et al. (2017) via FlyBase (https://flybase.org/). (B) FAIRE and RNA sequence analysis of the *Drosophila Smn* genomic region over embryonic development and in larval imaginal discs, data mined from McKay and Lieb (2013). (C) Transcriptomic analysis of SMN mRNA levels throughout *Drosophila* developmental stages, data mined from Graveley et al., 2011 via FlyBase (https://flybase.org/). (D) Workflow schematic of non-permissive-to-permissive temperature-shift experiments, switching larvae from 29°C to 22°C at 1 or 2 DPE. (E,F) Pupation (E) and eclosion (F) percentages of wild-type, Tudor domain mutant and YG box mutant (T205I) larvae raised exclusively at 22°C (blue), switched from 22°C to 29°C at L1 stage (yellow), switched from 22°C to 29°C at L2 stage (orange) or raised exclusively at 29°C (red) (150-420 larvae per genotype, 50 larvae per biological replicate). Error bars represent mean±95% c.i. Adjusted *P*-values calculated using Tukey's multiple comparisons test: ns, not significant (*P*>0.05); *P<0.05, **P<0.01, ***P<0.001). Sample size (number of larvae): WT 22°C (150), L1 (167), L2 (192), 29°C (394); F70S 22°C (150), L1 (233), L2 (255), 29°C (130); V72G 22°C (150), L1 (300), L2 (350), 29°C (290); G73R 22°C (150), L1 (278), L2 (293), 29°C (400); I93F 22°C (150), L1 (300), L2 (300), 29°C (150); T205I 22°C (150), L1 (345), L2 (300), 29°C (420).

As shown in Fig. 5A, developmental proteomic analysis demonstrates that levels of SMN protein are nearly 300-fold greater in embryos than during larval stages (Casas-Vila et al., 2017). Remarkably, chromatin accessibility (FAIRE-seq) analysis (McKay and Lieb, 2013) reveals that the *Smn* promoter region is essentially closed throughout embryonic development, suggesting its transcriptional quiescence (Fig. 5B). Indeed, transcriptomic (RNA sequence) profiling of the same embryos shows that *Smn* mRNA levels progressively decrease during embryogenesis (Graveley et al., 2011; Fig. 5B,C). We conclude that nearly all of the *Smn* mRNA and protein that is present in the animal during its first 24 h of life is maternally deposited. Western blot analysis of *Smn* null mutants during early larval development showed that the maternal contribution of SMN protein persists throughout the 1st larval instar (L1) and is essentially depleted by the 2nd (L2; Praveen et al., 2012). These data are consistent with the developmental proteomics (Fig. 5A), showing that SMN levels during L2, early L3 and late (wandering) L3 are at or below the limits of detection (Casas-Vila et al., 2017). Moreover, SMN levels in newly eclosed adults are also very low, but rise dramatically in 1 week old females (Fig. 5A). This observation suggests that the high levels of SMN detected in the older females is due to the maternal production of eggs.

Transgenic lines expressing either *Smn* TDMs or controls were initially raised at 29°C (non-permissive), then switched to 22°C (permissive) at 1 or 2 days post-egg laying (DPE) and viability was assessed (Fig. 5D). Somewhat surprisingly, the large maternal contribution of SMN appears to be sufficient for embryonic development, even at the non-permissive temperature (Fig. 5E,F). Note that because SMN is required for ovarian development (Lee et al., 2009), we are unable to generate animals that completely lack maternal SMN. However, females that are raised at permissive temperature and then switched to the non-permissive temperature for mating and egg-laying are able to produce viable offspring if the progeny are switched to permissive temperature at either L1 or L2 (Fig. 5F). Control TDM larvae maintained at 29°C for the entire time course failed to pupate (Fig. 5E). Thus, as suggested by the proteomic data (Fig. 5A), high levels of SMN do not appear to be required for progression from L1 to L3.

**Fig. 6.**
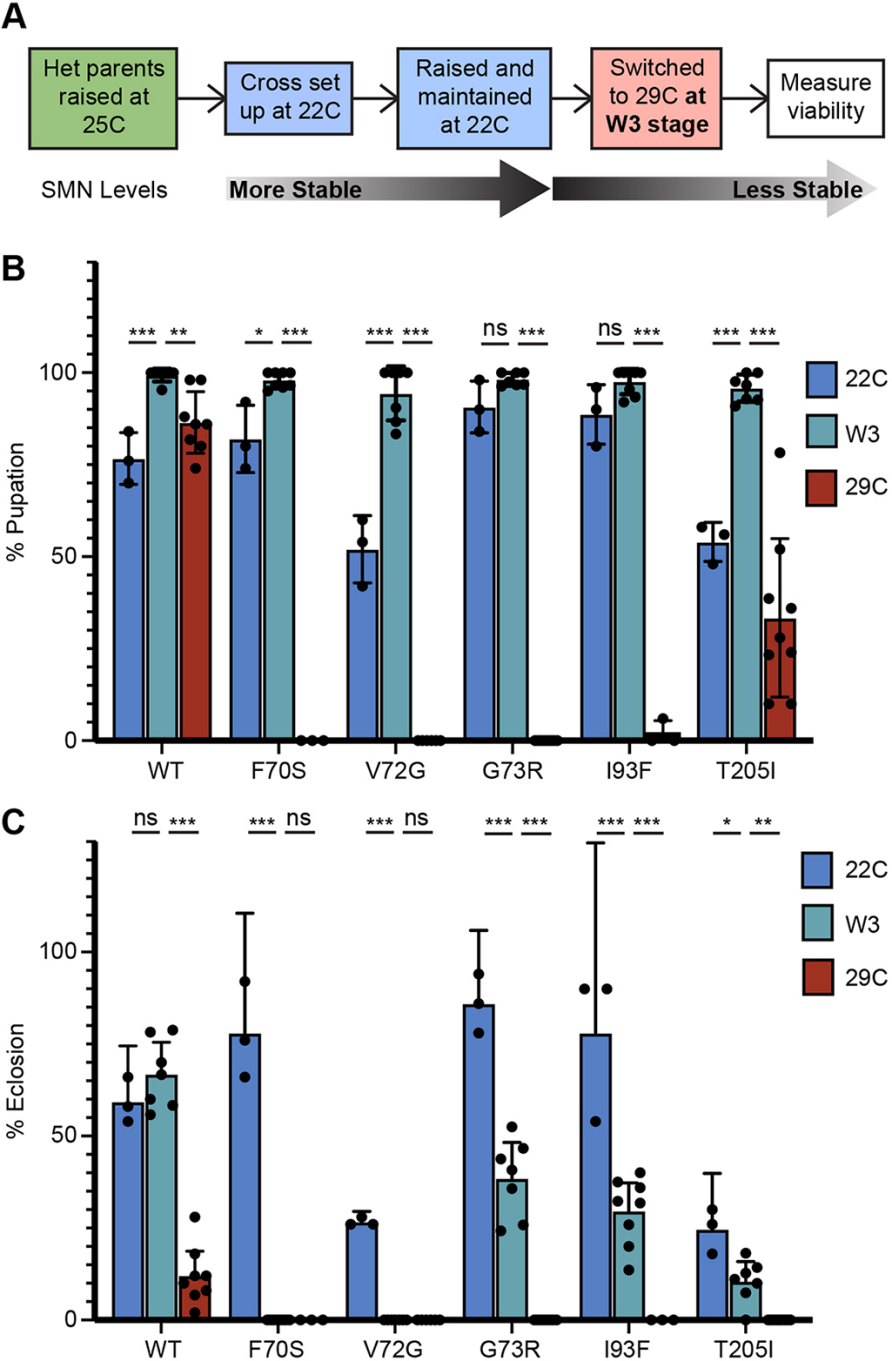
**Permissive-to-non-permissive temperature-shift viability assays during late larval and pupal development.** (A) Workflow schematic of permissive-to-non-permissive temperature-shift experiments, switching wandering 3rd instar (W3) larvae from 22°C to 29°C. (B,C) Pupation (B) and eclosion (C) percentages of wild-type, Tudor domain mutant and YG box mutant (T205I) larvae raised exclusively at 22°C (blue), switched from 22°C to 29°C at W3 stage (teal) or raised exclusively at 29°C (red); 150-420 larvae per genotype, ∼50 larvae per biological replicate. Error bars represent mean±95% c.i. Adjusted *P*-values calculated using two-way ANOVA and Tukey's multiple comparisons test: ns, not significant (*P*>0.05); *P<0.05, **P<0.01, ***P<0.001. Sample size (number of larvae): WT 22°C (150), W3 (220), 29°C (394); F70S 22°C (150), W3 (230), 29°C (130); V72G 22°C (150), W3 (176), 29°C (290); G73R 22°C (150), W3 (221), 29°C (400); I93F 22°C (150), W3 (206), 29°C (150); T205I 22°C (150), W3 (232), 29°C (420).

Interestingly, the expression of SMN rises dramatically in midpupation, only to drop again during later stages (Fig. 5A). In preparation for this second burst of activity during metamorphosis, the *Smn* promoter region is largely nucleosome-free by the time animals reach the wandering 3rd instar (L3) and remains open in the pharate adult (Fig. 5B). To determine whether high levels of SMN are required for larval progression, pupariation and eclosion, we carried out temperature-switch experiments. Progeny were initially raised at 22°C and then switched to 29°C after they reached the wandering 3rd instar (Fig. 6A). TDM larvae exposed to the non-permissive temperature at this later stage of development display normal pupation frequencies (Fig. 6B). Strikingly, however, eclosion frequencies of the TDM larvae were similar to those of larvae that had been raised exclusively at 29°C (Fig. 6C). A more thorough investigation found that most of the TDM pupae that died during pupation arrested late in pupal development, predominantly in the pharate stage (Fig. S2). These results demonstrate that although elevated SMN levels are not required for the earliest stages of larval development, high levels are required to complete metamorphosis.

### Baseline levels of SMN are required for normal adult longevity

**Fig. 7.**
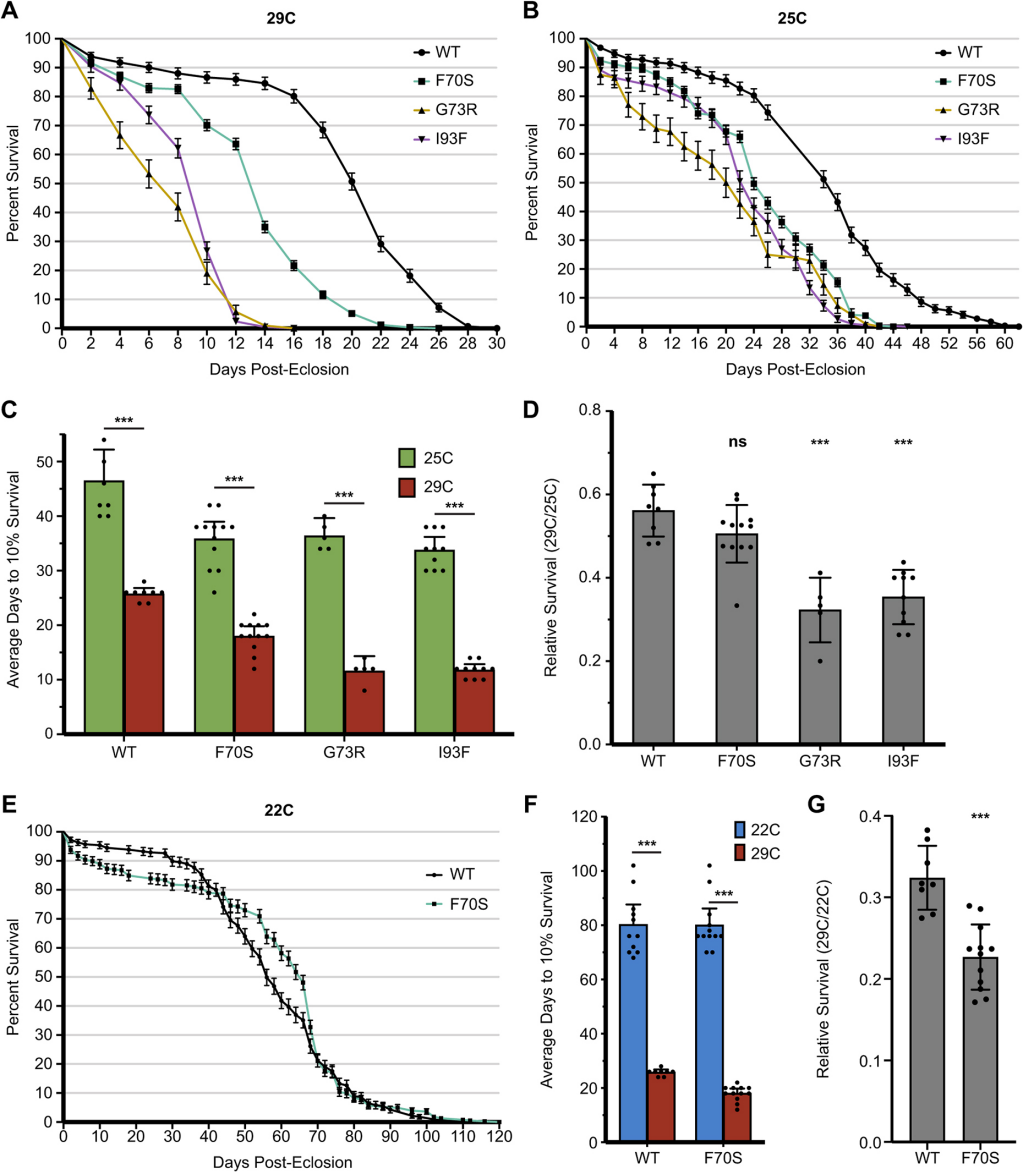
**Adult longevity of select SMN Tudor domain mutants at permissive and non-permissive temperatures.** (A,B) Survival plots of WT and Tudor domain mutant adult flies at 29°C (A) or 25°C (B). The flies were raised at 25°C (WT, G73R, I93F) or 22°C (F70S) prior to eclosion and then moved to the experimental temperature <24 h post-eclosion. The number of adults for each group ranges from 150 to 600. Live adults were counted every 2 days. Error bars represent mean±95% c.i. WT, black; F70S, green; G73R, yellow; I93F, purple. (C) Average time to reach 10% survival for adults at 29°C (red) and 25°C (green). Error bars represent mean±95% c.i. (D) Comparing relative longevity (10% survival 29°C/10% survival 25°C). Error bars represent mean±95% c.i. (E) Survival plot of WT (black) and F70S mutant (green) adult flies raised and maintained at 22°C. (F) Average time to reach 10% survival for adults raised at 22°C (blue) and 29°C (red). Error bars represent mean±95% c.i. (G) relative survival (10% survival 29°C/10% survival 22°C). Error bars represent mean±95% c.i. *P*-values for 10% survival were calculated using two-way ANOVA with Sidak's multiple comparisons test: ***P<0.001. *P*-values for longevity ratios were calculated using one-way ANOVA and Student's *t*-test: ns, not significant (*P*>0.05); ***P<0.001. Sample size (number of adults): WT 22°C (326), 25°C (289), 29°C (292); F70S 22°C (385), 25°C (578), 29°C (586); G73R 25°C (94), 29°C (105); I93F 25°C (196), 29°C (209).

Finally, we examined the effect of decreased SMN stability during adulthood. Studies in mice show that high SMN levels are not required for survival as adults; however, a baseline level of SMN is necessary for normal longevity (Sahashi et al., 2013). To test if the same trend holds in the fly, a subset of homozygous *Smn* transgenic stocks containing the WT, F70S, G73R or I93F transgenes (as described in the Materials and Methods section) was used. These lines were raised through embryonic, larval and pupal development at 25°C, with the exception of F70S, which had to be raised at 22°C in order to produce a testable number of adults. Within the first 24 h post-eclosion, adult animals were either maintained at 25°C or switched to 29°C. This paradigm allowed us to assess the effects on longevity when SMN protein is reduced exclusively during the adult stage. Adult longevity was measured by recording the number of surviving adults every 2 days post-eclosion (Fig. 7A,B). As expected all genotypes, including WT, showed reduced longevity at 29°C (Fig. 7A) compared with 25°C (Fig. 7B,C). However, the TDM lines that were switched to non-permissive temperature exhibited a significant drop in survival (dying within 6-16 days) compared with their permissive temperature counterparts (24-38 days) (Fig. 7A,B). To account for the baseline effects of elevated rearing temperature, we compared survivorship at 25°C and 29°C for each genotype (survivorship ratio=days to 10% survival at 29°C/days to 10% survival at 25°C). Importantly, when comparing relative survival at the 10% threshold, the survivorship ratio of the WT line was 56% (Fig. 7D), whereas the G73R and I93F TDMs displayed significantly reduced ratios of 32% and 35%, respectively (Fig. 7D).

The F70S mutant displayed a 51% survivorship ratio that was not significantly different from that of WT (Fig. 7D). This surprising result is possibly due to the fact that the F70S adults remain severely affected at 25°C (Fig. 1B,C), whereas the G73R and I93F defects at 25°C are mild. To account for this discrepancy, a second study was carried out at 22°C with the WT and F70S transgenic stocks (Fig. 7E). Unlike other culturing temperatures, the WT and F70S animals exhibit similar longevities at 22°C (Fig. 7F). When comparing the relative survival of these genotypes between 29°C and 22°C, there is a statistically significant difference between WT (32%) and F70S (23%) (Fig. 7G). F70S adults also displayed a sex-specific difference in longevity that was not observed in the WT animals (Fig. S3). These data indicate that the TDMs exhibit a differential sensitivity to temperature even after development is complete, and that an above-baseline level of SMN is needed well into adulthood. In conclusion, these temperature-sensitive TDM lines should prove useful in future studies of the molecular, physiological and behavioral consequences of SMN loss in a developing, free-living organism.

## DISCUSSION

In this study, a series of point mutations within the SMN Tudor domain were found to exhibit pronounced temperature sensitivity relative to the wild type or YG box mutant SMN proteins. This differential sensitivity leads to significantly reduced SMN protein levels at higher temperatures. We utilized this paradigm to assess the effects of temporally manipulating SMN protein levels across various developmental time points.

### Effects of Tudor domain mutations on SMN stability

SMN protein levels are the strongest known modifiers of SMA disease severity, and small changes in these levels can dramatically affect age-of-onset and symptomatic severity. The relationship between SMN levels and SMA severity can be described using a two-threshold model (O'Hern et al., 2017). At levels above the upper threshold of SMN protein, individuals are unaffected. Below the second (lower) threshold, organisms die very early in development. Between these two thresholds of SMN expression is a region termed the ‘SMA zone’, wherein small changes in SMN levels cause large changes in disease severity (O'Hern et al., 2017).

Here, we show that single-residue substitutions in the SMN Tudor domain directly affect SMN protein stability *in vivo*. Our data show that SMN protein production is also reduced in these cases, which could contribute to overall SMN levels. These findings have important implications for the effects of similar mutations on SMA patients. In *Drosophila*, TDMs exhibit reduced SMN levels, leading to defects in organismal viability, locomotion and lifespan when animals are raised at higher temperatures (27°C and 29°C). This idea is consistent with data from SMA patients, suggesting that certain TDMs lead to reduced SMN protein levels in human cells (Takarada et al., 2017). Indeed, the phenotype of the *Drosophila* F70S mutant at the non-permissive temperature more accurately aligns with that of the corresponding human *SMN1* missense mutation, W92S, which causes Type 1 SMA (Kotani et al., 2007). Given that the *Drosophila* TDMs show significant protein reduction and instability at 27°C and 29°C, respectively, it is possible that the thermoregulated human internal temperature of 37°C is enough to destabilize SMN protein containing these patient mutations and cause disease phenotypes. Thus, the acute sensitivity of the TDMs to small shifts in temperature highlights a previously unrecognized variable in SMN biology.

Comparing our phenotypic data with the SMN Tudor domain structure sheds light on the relative importance of specific regions of the protein to its overall stability. Previous studies implicated the YG box in regulating SMN protein levels; however, this is now thought to occur via a completely distinct mechanism. That is, self-oligomerization of SMN leads to sequestration of a ubiquitin-dependent degron motif located within the SMN C-terminal region (Cho and Dreyfuss, 2010; Gray et al., 2018). The only Tudor domain mutation we assayed that does not display differential temperature sensitivity is Y107C. Unlike the other TDMs we tested, Y107C is located within the dimethylarginine-binding ‘pocket’ of the Tudor domain (Tripsianes et al., 2011), and probably affects the ability of SMN to bind Sm proteins and other potential targets.

One outstanding question is how biochemical/biophysical properties of these TDMs affect protein stability. It is likely that some or all of these mutations cause misfolding of the normal tertiary structures within the Tudor domain (Fig. 4D). Previous *in vitro* and *in silico* studies showed that certain TDMs lead to misfolding and decrease stability (Hossain and Hosen, 2019; Li, 2017; Sneha et al., 2018; Tripsianes et al., 2011). Our own modeling shows that most SMN TDMs cluster around the same structural motif and cause a steric clash within this region when mutated (Fig. 4E). It is also possible that SMN instability is due to a loss of stabilizing interactions with binding partners, or a combination of both. However, our findings represent the first *in vivo* studies to measure the relative stability of SMN TDMs. Moreover, our experimental system allows us to test the function of an individual SMN mutant in the absence of wild-type SMN. These findings could prove important when developing treatments aimed at increasing SMN levels in patients. For example, small molecules targeting the Tudor domain could potentially improve SMN stability and increase protein levels at the post-translational level.

### Temporal requirement for high SMN levels across development

In addition to uncovering a second disease mechanism, the discovery of temperature-sensitive SMN alleles provides a new genetic tool for the *in vivo* study of SMA-related phenotypes. Here, we have used this temporally manipulatable system to test the requirements for high levels of SMN during *Drosophila* development. By manipulating the timing of exposure to permissive and non-permissive temperatures, we observed that production of high SMN levels is not required for larval development (1-2 DPE). Note that a baseline level of SMN is still required during these stages, as we and others have reported that *Smn* null animals undergo an early developmental arrest (Garcia et al., 2013; Shpargel et al., 2009).

We found that high SMN levels are crucial between the wandering 3rd instar larval stage and the end of pupation. This finding correlates with whole-organism proteomic data showing that SMN levels are extremely high during both embryonic and pupal stages of *Drosophila* development (Casas-Vila et al., 2017). Interestingly, both embryonic and pupal stages involve the production of a new free-living animal. That is, embryogenesis results in a larva and metamorphosis involves a near complete regrowth of the body into an adult animal. As such, *Drosophila* pupal development more closely resembles perinatal development in mice and humans. Our results show that it might be more medically relevant to directly compare developmental stages in which the majority of tissue formation and differentiation takes place. In humans, this would correspond to the prenatal and perinatal stages of development, when, indeed, SMN levels are highest (Ramos et al., 2019). Thus, in terms of translational medicine, pupariation is perhaps a more appropriate stage of development on which to focus future studies of neuromuscular development in *Drosophila*.

### Temperature-sensitive SMN mutants as a novel genetic tool for studying SMA

Temperature-sensitive SMN mutations can be utilized as a system to further interrogate the effects of SMN protein level homeostasis at different stages of development. The ability to control SMN protein levels *in vivo* using temperature allows us to more easily test molecular, physiological and behavioral effects of reduced SMN levels during later developmental stages that were previously difficult to study because few individuals reached that stage. Similarly, we now have the ability to quickly rescue SMN protein levels simply by switching culturing temperatures. Altering SMN levels will enable studies of various longitudinal effects of SMN rescue at different points in development.

This model also provides a tool to screen for other factors involved in SMN biology and disease etiology. The TDM stable stocks can be successfully maintained at lower temperatures (22°C) and have the advantage of having no WT SMN, unlike the parental animals in a cross. These stable stocks are also beneficial because raising their culturing temperature can produce a situation where the majority of individuals die just before pupation or eclosion. When crossed to mutants of candidate genes or deficiency lines at these higher temperatures, any change in viability would be relatively easy to distinguish and signal a potential genetic interaction with *Smn*.

In conclusion, we have discovered a domain-specific effect on SMN stability that affects SMN protein levels, motor function and viability in *Drosophila* models of SMA. Further study of the Tudor domain and its role in the stability of SMN protein might be an important avenue for developing future SMA treatments that are effective in combination with existing therapies. The development of this temperature-sensitive model of SMA in *Drosophila* has allowed us to uncover the crucial development time points for high SMN levels. In the future, this system could be used to further elucidate important functions of SMN during these stages and to screen for novel disease interactors and pathways.

## MATERIALS AND METHODS

### Fly lines and husbandry

Balanced patient mutation lines (*Smn^X7^,* *Smn^TG^*/TM6B-GFP) were generated as described (Praveen et al., 2012) where ‘TG’ represents one of 14 *Smn* transgenes. Briefly, the lines were generated using ΦC31 integration at an insertion site located in chromosome band position 86F8. The *Smn* transgenic construct is a ∼3 kb fragment containing the entire *Smn* coding region, expression of which is driven by the native *Smn* promoter. The transgene also contains an N-terminal 3X-FLAG tag that was used in this study to visualize SMN protein, circumventing the potential differences in α-SMN antibody binding between mutant forms of SMN. The *Smn^X7^* and *Smn^D^* alleles are previously described null alleles (Chang et al., 2008; Rajendra et al., 2007), and both stable stocks are GFP balanced. To generate single-copy transgenic mutants (*Smn^X7^,* *Smn^TG^/Smn^X7^*) for the viability, locomotion, western blot and developmental timing assays, *Smn^X7^*/TM6B-GFP virgin females were crossed to *Smn^X7^, Smn^TG^*/TM6B-GFP males at the desired temperature (18°C, 22°C, 25°C, 27°C or 29°C). Crosses were performed on molasses-based agar plates with yeast paste, and then GFP-negative larvae were sorted into vials containing standard molasses fly food at the 2nd instar larval stage to prevent competition from heterozygous siblings.

The stable wild-type (WT) and mutant (F70S, G73R and I93F) lines used in the longevity assays were generated by crossing *Smn^D^*/TM6B-GFP virgin females to *Smn^X7^, Smn^TG^*/TM6B-GFP males at room temperature. Progeny lacking the balancer chromosome were then allowed to propagate and develop into stable lines.

The WT and F70S lines used in the CHX experiments were stable stocks homozygous for the transgene. These stocks were generated by crossing males from the variable copy number stocks above to *Smn^X7^, Smn^TG^*/TM6B-GFP virgin females and then sorting against the balancer chromosome markers.

All of the stocks except for the stable patient mutation lines were raised and maintained in a 25°C incubator unless being used for an assay. The stable patient mutation lines were raised and maintained at room temperature unless being used for an assay. Experimental temperatures for the assays were maintained using 18°C, 22°C, 25°C, 27°C and 29°C incubators. All stocks were maintained in bottles containing standard molasses fly food.

### Viability assays

To assess viability, crosses were maintained and progeny were raised at the desired temperature on molasses agar plates. A total of 25-50 GFP-negative progeny at the late 2nd to early 3rd instar stages were sorted into vials containing standard molasses fly food. After sufficient time had passed, pupal cases were counted and marked and any adults were counted and removed from the vial. Any new pupal cases or adults were recorded every 2 days. The percentage viability was calculated at both the pupal and adult stages. Pupal viability (percentage pupation) was calculated by dividing the number of pupal cases by the initial number of larvae and multiplying by 100 (# pupae/# initial larvae×100). Adult viability (percentage eclosion) was calculated similarly, but using the number of adults as the numerator (# adults/# initial larvae×100). Each vial was considered a biological replicate in respect to calculating averages and standard error.

### Larval locomotion assays

To assess the motor function of larvae at permissive and non-permissive temperatures, crosses were maintained and progeny were raised at the desired temperature. Once the larvae reached wandering 3rd instar larval stage, one to five larvae were placed onto the locomotion stage (a large molasses plate) at room temperature. The stage was then placed into a recording chamber to control light and reflections on the stage. Once all larvae were actively crawling, movement was recorded for at least 62 s on an iPhone6 at minimum zoom. Two recordings were taken for each set of larvae. At least 30 larvae were recorded for each experimental group. Locomotion videos were transferred to a PC and converted to raw video .avi files using the ffmpeg program. Videos were then opened in Fiji/ImageJ (https://imagej.net/Fiji), trimmed to ∼60 s of video and converted into a series of binary images. The wrMTrck plugin for ImageJ (http://www.phage.dk/plugins/wrmtrck.html) was used to analyze the video and determine larval size, average speed of movement and average speed normalized to larval size (BLPS) (Brooks et al., 2016). Each larva was treated as an individual when calculating average and standard error.

### SMN western blot analysis

To measure SMN protein levels at different temperatures, eight to 12 wandering 3rd instar larvae were collected per sample, snap frozen in a dry-ice ethanol bath and stored at −80°C. Each sample was considered a biological replicate. Larval samples were then homogenized in RIPA buffer and 10× protease inhibitor cocktail [Halt™ Protease Inhibitor Cocktail (100×), Thermo Fisher Scientific] with a micropestle and spun at 13,000 rpm at 4°C to separate the soluble phase. The supernatant was transferred to a new microcentrifuge tube and spun again to separate any lipid phase. The protein samples were then quantified using Bradford assay (BioPhotometer, Eppendorf) and western samples were prepared with 50 µg protein and 1× SDS loading buffer then denatured in a 95°C heat block for 5 min.

Western samples were loaded and run in Mini-PROTEAN TGX Stain-Free Gels (Bio-Rad) at 300 V for 15 min (Mini PROTEAN^®^ Tetra Cell, Bio-Rad). The total protein marker was UV activated for 105 s (Fisher Biotech 312 nm Transilluminator) and then the gel was placed in the transfer cassette (XCell II™ Blot Module, Novex^®^ Life Technologies™) and transferred onto low-fluorescence polyvinylidene difluoride (PVDF) membrane (Immun-Blot^®^ LF PVDF Membrane Roll, Bio-Rad). After the transfer, total protein on the membrane was imaged using UV exposure with an Amersham Imager 600 (GE Healthcare). The membrane was then blocked in 5% milk [in Tris-buffered saline with Tween 20 (TBST)] for 1 h at room temperature with gentle shaking, then incubated with α-FLAG horseradish peroxidase (HRP)-conjugated primary antibody [1:10,000 in TBST; Monoclonal ANTI-FLAG^®^ M2-Peroxidase (HRP) antibody produced in mouse, Sigma-Aldrich, cat. #A8592] overnight at 4°C. The next day the membrane was washed three times for 5 min in TBST at room temperature, then incubated with detection reagent (Amersham ECL™ Prime Western Blotting Detection Reagents, GE Healthcare) for 5 min. The chemiluminescence was detected and imaged using an Amersham Imager 600 (GE Healthcare). The FLAG-SMN levels and total protein were quantified using the ImageQuant TL 8.1 (1D analysis) program. Any samples with low-quality total protein signal were excluded from the analysis. Averages and standard error were determined based on the biological replicates for each condition. Any outliers were determined using the Grubbs' test (https://www.graphpad.com/quickcalcs/grubbs1/) and removed from the data set before analysis.

### Protein stability assays

To assess the stability of SMN protein, CHX treatment was applied to wandering 3rd instar larvae. Larvae were produced from crosses and contained a single copy of the *Smn* transgene. Crosses and progeny were maintained at 22°C, where embryos were laid onto molasses-based agar plates. Larvae of the desired genotype were sorted into molasses food vials. When the larvae reached the wandering 3rd instar developmental stage, they were dissected open to expose all the internal and external tissues to treatment media. The simple dissection was performed using dissecting tweezers to peel back a strip of the larva's exoskeleton. Five larvae were dissected for each sample. The control treatment contained 5 µg/µl puromycin [Sigma-Aldrich, puromycin dihydrochloride from *Streptomyces alboniger* (P7255), stock solution of 25 mg/ml dissolved in autoclaved water] in Schneider's *Drosophila* medium (1×) (Gibco, 21720-024). The experimental treatment contained 5 µg/µl puromycin and 100 µg/ml CHX (Sigma-Aldrich, C7698, stock solution of 10 mg/ml dissolved in autoclaved water) in Schneider's medium. Larvae were placed in a microcentrifuge tube containing 500 µl of treatment, and then incubated with the treatment for the desired time (hours) at 29°C. After the desired treatment time, medium was removed and sample preparation, protein extraction and western blot analysis were performed as described above. The α-puromycin antibody was used at a concentration of 1:1000 (Kerafast, #EQ0001) in TBST, followed by an α-mouse secondary antibody at a concentration of 1:5000 (Pierce, PI31430) in TBST.

### Temperature-switch assays

To assess viability after exposure to permissive and non-permissive temperatures, SMN mutant larvae were switched between 22°C and 29°C at different developmental stages. The temperature-switch assays in Figs 5 and 6 were performed with crosses, wherein mutant progeny contained one copy of the *Smn* transgene and a maternal component of wild-type SMN.

During the non-permissive-to-permissive viability assays, crosses and progeny were maintained and raised at 29°C. Embryos were laid on molasses-based agar plates within a 4 h time window then, either 1 or 2 DPE, the molasses plates were moved from 29°C to 22°C. Within 36 h of being switched to 22°C, mutant larvae of the desired genotype were separated from their siblings and placed in a vial of molasses food (∼50 larvae/vial). Viability was then assessed by counting the number of pupae (percentage pupation) and adults (percentage eclosion) in each vial compared with the number of larvae as described above. Each vial was treated as a separate replicate and was used to calculate averages and standard error.

During the permissive-to-non-permissive viability assays, the parental generation and progeny were raised at 22°C with the same molasses plate system and timed temperature switches as described above; the single difference was that developing larvae were moved from 22°C to 29°C. In the wandering 3rd instar larvae temperature switch, progeny remained at 22°C and larvae were sorted into a molasses food vial while still at 22°C. Once the larvae began to wander, each larva was transferred to a new vial at 29°C (∼50 larvae/vial). Viability was assessed with the same method as above.

### Longevity assays

To assess longevity at permissive and non-permissive temperatures, newly eclosed adults (less than 24 h post-eclosion) were collected and put into fresh vials of molasses food. Males and females were separated into different vials. Each vial contained ten or fewer adults to reduce stress and crowding. Around half of these adults remained at the permissive temperature (25°C or 22°C) and the other half were switched to a non-permissive temperature (29°C). Animals were transferred to a fresh vial two to three times per week to prevent death owing to suboptimal food conditions. The number of surviving adults was recorded on the collection day and then every 2 days until all adults had expired. Any adults that were injured/killed or escaped during the experiment were removed from the counts. Every 20-50 adults were considered a biological replicate when determining averages, standard error and survival thresholds.

### Structural modeling

A model of *D. melanogaster* SMN (dmSMN) was generated using HHpred2 (Zimmermann et al., 2018). The template used was the Tudor domain from human SMN1 (PDB ID 4qq6) (Liu et al., 2019 preprint). Figures of the 4qq6 structure and the dmSMN model were rendered in PyMOL (PyMOL The PyMOL Molecular Graphics System, Version 2.0 Schrödinger, LLC). A dimethylated arginine was placed in the active site of the 4qq6 and dmSMN structures based on the solution structure of the complex of human SMN with the dimethylated arginine ligand (PDB ID 4a4e) (Tripsianes et al., 2011).

### Statistical analysis

All graphing and statistical calculations were performed using GraphPad Prism (Version 8.2.0). All organismal viability, SMN protein levels (variable temperature and CHX) and temperature-switch experiments were analyzed using a two-way ANOVA with Tukey's multiple comparison test (α=0.05). Larval locomotion was analyzed using unpaired multiple *t*-tests (α=0.05). Adult longevity at 10th percentile was analyzed using a two-way ANOVA with Sidak's multiple comparisons test (α=0.05). The 25°C versus 29°C relative survival was analyzed using ordinary one-way ANOVA with Dunnett's multiple comparisons test (α=0.05). The 22°C versus 29°C relative survival was analyzed using Welch's *t*-test (α=0.05). In all graphs, error bars are expressed as the mean±95% c.i.

## Supplementary Material

Supplementary information
