# Supplementary material for: Temperature-sensitive spinal muscular atrophy-causing point mutations lead to SMN instability, locomotor defects and premature lethality in Drosophila
Source: Dis Model Mech. 2020 May 22;13(5):dmm043307. doi: 10.1242/dmm.043307 (PMC7325441; doi:10.1242/dmm.043307)
Supplement: Supplementary information [file dmm-13-043307-s1.pdf]

Supplemental Figure 1

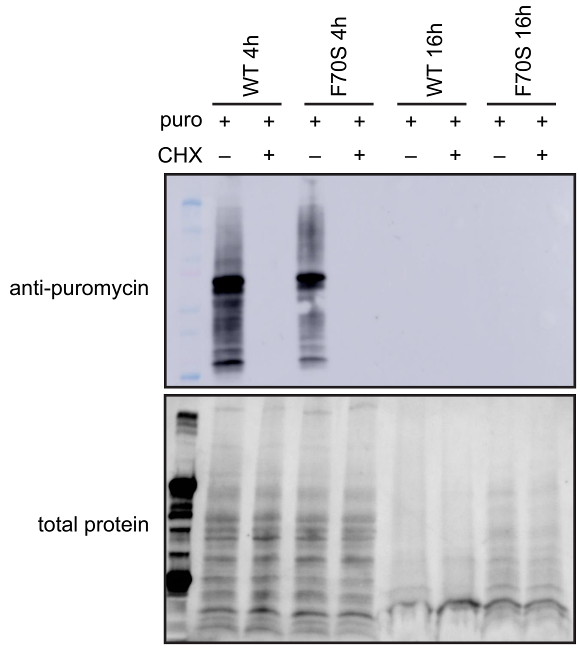

Figure S1. α-puromycin western blot in the presence and absence of cycloheximide (CHX). Western blot of wandering 3rd-instar wildtype larvae (10 larvae/sample) after puromycin treatment with or without CHX (4-hour or 16-hour treatment). Lower panel is total protein visualized using fluorescence as described in Materials and Methods. Upper panel is western blot of puromycin, which represents newly translated protein.

Disease Models & Mechanisms • Supplementary information

Supplemental Figure 2

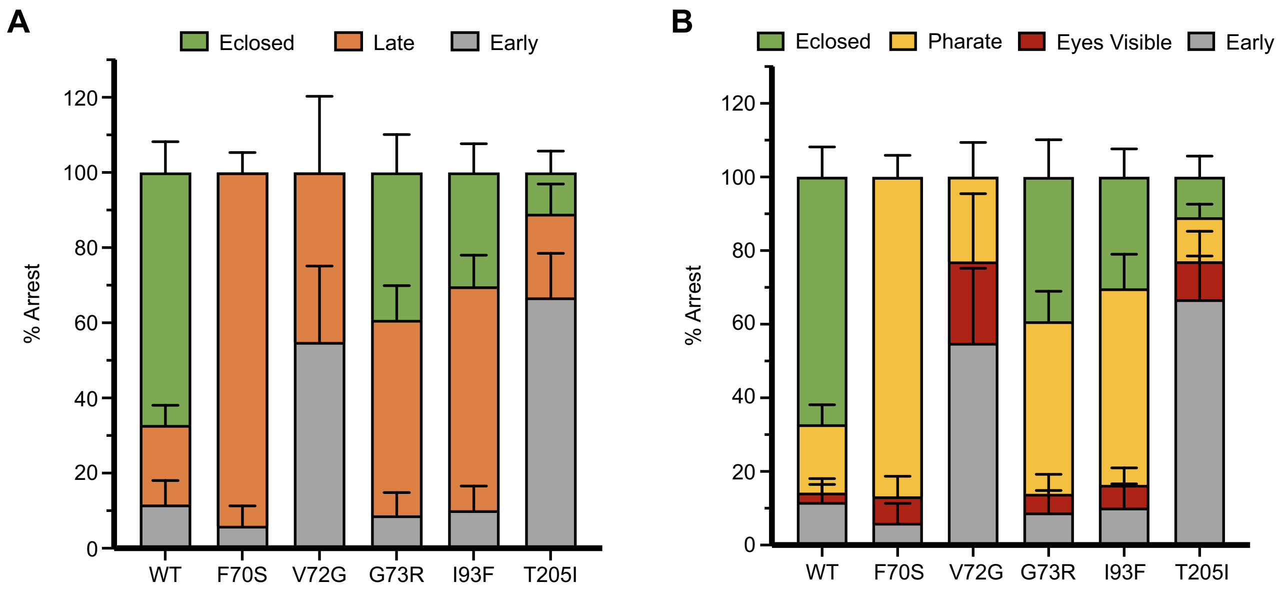

Figure S2. Developmental arrest of Tudor mutants during permissive-to-nonpermissive temperature switch. (A) Graph depicting the percentage of pupae that arrest early in pupal development (grey), late in pupal development (orange), or fully eclose into adults (green) for WT, TDMs, and T205I transgenic animals when W3 larvae are switched from 22ºC to 29ºC. (B) Same data as in (A), but the "Late" category has been separated further into "Eyes Visible" (red) and "Pharate" (yellow). Error bars represent mean±95% c.i. Sample size (# of larvae) – WT: 218, F70S: 226, V72G: 165, G73R: 217, I93F: 201, T205I: 224.

Disease Models & Mechanisms • Supplementary information

Supplemental Figure 3

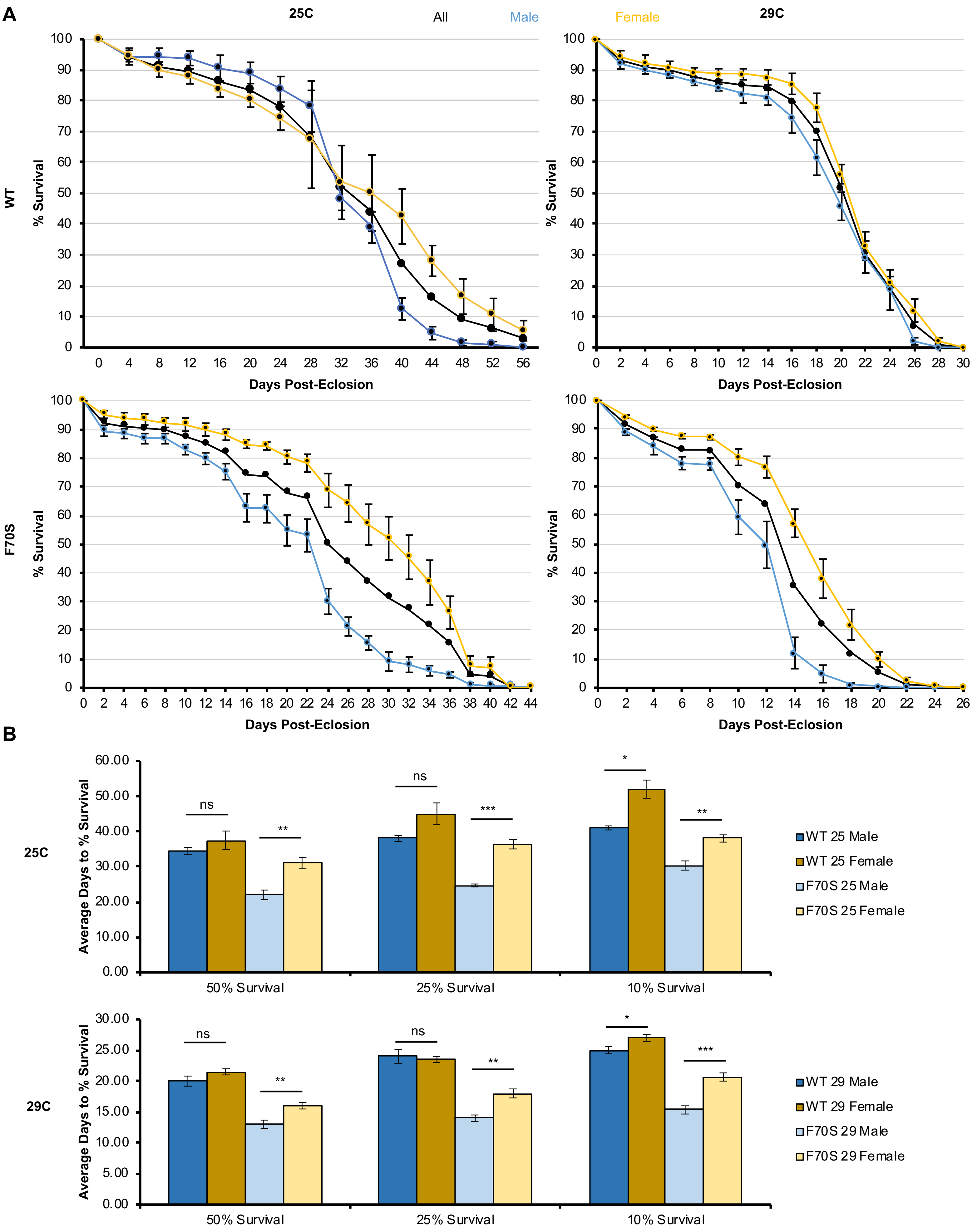

Figure S3. Sex-specific adult longevity. (A) Survival plots of WT and F70S Tudor domain mutant adult flies at 29°C (left) or 25°C (right). Number of adults for each genotype-temperature combination range from 140 to 300. WT survival plots are on top, F70S survival plots are one bottom. Live adults were counted every two days. Error bars represent standard error. Combined (Male+Female)-black, Male-blue, Female-yellow. (Left) WT 25°C flies were raised at 25°C and were shifted to a new vial remaining at 25°C until death; F70S flies were raised at 22°C and shifted to 25°C after eclosion. (Right) WT 29°C flies were raised at 25°C (F70S flies were raised at 22°C) and then both genotytpes were shifted to a new vial at 29°C less than 24h after eclosion until death (B) Comparing days to 50%, 25%, or 10% survival or less between male (blue) and female (yellow) adults at 25°C (above) or 29°C (below). Darker bars represent WT adults, lighter bars represent F70S adults. Error bars represent mean±s.e.m. P-values calculated using student's t-test (* $p<0.05$, ** $p<0.01$, *** $p<0.001$). Sample size (# of adults) – **WT 25ºC** Male: 140, Female: 149; **WT 29ºC** Male: 142, Female: 150; **F70S 25ºC** Male: 290, Female: 288; **F70S 29ºC** Male: 290 Female: 296.
